# Adolescents’ prospective screen time by gender and parental education, the mediation of parental influences

**DOI:** 10.1186/1479-5868-10-89

**Published:** 2013-07-06

**Authors:** Torunn H Totland, Mona Bjelland, Nanna Lien, Ingunn H Bergh, Mekdes K Gebremariam, May Grydeland, Yngvar Ommundsen, Lene F Andersen

**Affiliations:** 1 Department of Nutrition, University of Oslo, P.O. Box 1046 Blindern, NO-0316 Oslo, Norway; 2 Department of Coaching and Psychology, Norwegian School of Sport Sciences, P.O. Box 4014 Ullevål stadion, NO-0806 Oslo, Norway; 3 Department of Sports Medicine, Norwegian School of Sport Sciences, P.O. Box 4014 Ullevål Stadion, 0806 Oslo, Norway

**Keywords:** Television, Computer games, Socioeconomic position, Parental modelling, Parental regulation, Gender dyads

## Abstract

**Background:**

The present study investigated associations in gender dyads of parents’ and adolescents’ time spent on television and video viewing (TV/DVD), and computer and electronic game use (PC/games) at the ages of 11 and 13 years. Possible mediating effects of parental modelling and parental regulation in the relationship between parental education and adolescents’ prospective TV/DVD and PC/game time were further examined.

**Methods:**

A total of 908 adolescents, participating at both ages 11 and 13 years in the Norwegian HEalth In Adolescents (HEIA) cohort study (2007–2009), were included in the analyses. Data on adolescents’, mothers’ and fathers’ self reported time spent on TV/DVD and PC/games were measured at both time points by questionnaires. Correlation coefficients were used to examine gender dyads of parents’ and adolescents’ reports. Mediation analyses using linear regression investigated possible mediation effects of parental modelling and parental regulation in the prospective relationship between parental education and adolescents’ time spent on TV/DVD and PC/games between the ages of 11 and 13 years.

**Results:**

Correlations of screen time behaviours in gender dyads of parents and adolescents showed significant associations in time spent on TV/DVD at the age of 11 and 13 years. Associations between mothers and sons and between fathers and daughters were also observed in time spent on PC/games at the age of 11 years. Maternal and paternal modelling was further found to mediate the relationship between parental education and adolescents’ prospective TV/DVD time between the ages of 11 and 13 years. No mediation effect was observed for parental regulation, however a decrease in both maternal and paternal regulation at the age of 11 years significantly predicted more TV/DVD time among adolescents at the age of 13 years.

**Conclusion:**

Cross-sectional and longitudinal relationships were observed in gender dyads of parents’ and adolescents’ screen time behaviours at the ages of 11 and 13 years, and further studies including both parents and their children should be emphasized. Moreover, maternal and paternal modelling were found to be important target variables in interventions aiming to reduce social differences by parental education in adolescents’ prospective time spent on TV/DVD.

## Background

Sedentary behaviour is one of the energy balance related behaviours found to be associated with body weight, overweight and obesity in children and adolescents [1-8], and is emerging as an important issue in public health [9,10]. Children and adolescents’ sedentary behaviours are shown to continue into adulthood [11], and time spent sedentary during childhood is thus associated with several health consequences as adults [12]. The age of 10–11 years is considered to be a key transition phase in a prevention perspective [13], and good opportunities for future health may be established among children growing into adolescence [14].

Sedentary behaviours can be defined as low-energy expenditure activities [5,9,15], and the most common sedentary behaviours are related to desk-based work and education, motorized transport, sitting while socializing and screen time [5,16]. Early adolescence is considered a critical time period for the onset of screen time behaviours [14,17]. European children and adolescents’ are in general found to exceed the recommendation of spending less than two hours on screen time a day [18-20]. Several reviews have therefore investigated correlates of sedentary behaviours [4,5,21,22] and screen time [23,24] in young people. However, insufficient evidence was found for prospective determinants of children and adolescents’ sedentary behaviours [22], and few investigated other screen time behaviours than television viewing. Reviews of cross-sectional studies found boys, older children, a higher body weight, lower socioeconomic status, lower parental education, non-white ethnicity and children living in one-parent families to be socio-demographic correlates of increased screen time among children and adolescents [5,21,23,24].

A previous cross-sectional study found the family television environment to partly mediate the relationship between maternal education and children’s television viewing [25]. Important factors in the home environment related to children’s and adolescents’ screen time are access to televisions and computers at home [5,26], parental screen time [22,24,26] and parental rules/regulation/limitation [5,26]. Little is known about gender specific relationships between parents and their children’s screen time [27]. Two cross-sectional and one longitudinal study were found to investigate the association between adolescents’ television viewing and parental television viewing [28-30] and parental rules of television viewing [29] with reports from mothers and fathers separately. Moreover, few cross-sectional and no longitudinal studies were found to specifically investigate the association between adolescents’ time spent on computer and electronic games with parental screen time behaviours [31,32] or parental screen time rules [31,33,34]. Longitudinal studies are currently needed to examine the relationship between the home environment and adolescents’ sedentary behaviours [26,27].

In the HEalth In Adolescents (HEIA) cohort study it has previously been reported that time spent on television and video viewing (TV/DVD) and on computer and electronic games (PC/games) increased significantly among boys and girls from the age of 11 to 13 years [35]. Demographic factors related to adolescents’ total screen time between the ages 11 to 13 years were lower parental education among girls and not living in two-parent families among boys [35]. Determinants in the home environment may be important target variables to consider in interventions aiming to reduce adolescents’ prospective screen time, and thus explain some of the educational differences in adolescents screen time. The purpose of the present paper was to investigate associations in gender dyads of parents’ and adolescents’ time spent on TV/DVD and PC/games, and to examine whether parental modelling and parental regulation mediate the relationship between parental education and TV/DVD or PC/game time from the age of 11 to 13 years.

## Methods

### Subjects and study design

The HEIA cohort consists of students from the 25 control schools of the randomized controlled intervention study HEIA. A total of 37 schools participated in the HEIA study, out of 177 schools identified from the largest towns/municipalities in seven counties surrounding the capital of Norway, with at least 40 pupils enrolled in 6th grade. These were randomly selected into 12 intervention schools and 25 control schools. The design and methodology of the HEIA study is described in detail elsewhere [36]. The present study was conducted according to the guidelines laid down in the Declaration of Helsinki and all procedures were approved by the Regional Committee for Medical Research Ethics and the Norwegian Social Science Data Service. Written informed consents were obtained from the parents of the included adolescents, and participation in the study was voluntary among both adolescents and parents at all times.

All 6th graders enrolled in the 25 control schools were invited to participate, resulting in a cohort sample of 1381 adolescents. At the baseline survey (T0) in September 2007, 975 (71%) adolescents participated. In May 2008, 970 (70%) attended the first follow up (T1), and 945 (68%) adolescents participated in the 20 months follow up (T2) during May 2009. Parents or legal guardians (hereafter called parents) of the adolescents were also asked to participate in the study at T0 and T2. For the purpose of the present paper, only participants attending T0 and T2 were included, resulting in 908 (93%) of the adolescents participating at T0. When including mothers’ and fathers’ reports at T0, the study sample was reduced to 738 (76%) and 630 (65%) respectively.

### Data collection

Information on both parents’ education was reported by parents on the adolescents’ consent forms. Internet-based questionnaires were filled in by the adolescents during school hours at each time-point, taking about 45 minutes to complete. The questionnaires assessed dietary, sedentary and physical activity behaviours and their determinants. Trained staff was available for questions during data collection at all times, and for measuring adolescents’ anthropometrics by height, weight, hip and waist at T0 and T2. Adolescents brought home separate questionnaires for their mothers and fathers. Both parents answered the same questions assessing dietary, sedentary and physical activity behaviours, their determinants as well as determinants related to their child’s behaviours at T0 and T2, taking about 45 minutes to complete. The questionnaires were returned to the schools in sealed envelopes by the adolescents, and then collected by the HEIA project workers. All questionnaires consisted of mostly pre-coded answer categories, in addition to some open ended fields with the possibility of specifying the responds.

### Outcome measures of screen time

Adolescents’ TV/DVD time was measured by a question modified from the PEACH study [37]. For the purpose of the present study, time used at weekdays and weekends were assessed separately, as previously recommended [38]. The question assessing TV/DVD time was phrased: “How many hours do you usually watch television and/or video on a normal weekday?” The same question was asked for a normal weekend day or day off from school. The answer categories were (recoding in brackets): half an hour (0.5), one hour (1), two hours (2), three hours (3), four hours (4), five hours or more (5). A similar question was composed to assess adolescents’ usual time spent on PC/games: “How many hours do you usually spend on computer, TV games or other electronic games on a normal weekday?” The same question was asked for a normal weekend day or day off from school. The answer categories were (recoding in brackets): no time (0), half an hour or less (0.5), one hour (1), two hours (2), three hours (3), four hours or more (4). Separate weekly scores for TV/DVD and PC/game time were calculated by summing hours reported for an average weekday (multiplied by five) and average weekend day (multiplied by two). The weekly outcome measures obtained acceptable test-retest correlation (Spearman’s rho=0.66-0.73) [35,36].

### Parental measures

Parental education was dichotomized into categories of 12 years or less and 13 years or more (equals university or college attendance). The parent with the longest education was used, or else from the one available. Parental behaviours (parental modelling) were measured by both parents as usual time spent on TV/DVD and PC/games by the same questions and pre-coded answer categories as for adolescents. However, in the parental questionnaires it was stated that the reported PC/game time was to be outside working hours. Parental regulation of TV/DVD was measured by mothers and fathers by a question derived from a previously reliability tested and published instrument [30]: “I restrict how much time my child spends watching TV”. A similar question was composed to measure parental regulation of PC/games: “I restrict how much time my child spends on computer and other electronic games”. The answer categories were based on a five point Likert scale (recoding in brackets): totally disagree (1), partly disagree (2), neither agree nor disagree (3), partly agree (4), totally agree (5).

### Correlates

Adolescents’ height and weight were measured objectively by project workers to the nearest 0.1 cm and 0.1 kg [39]. Age and gender specific body mass index cut-off values as proposed by the International Obesity Task Force was used to categorize the adolescents’ weight status as normal weight or overweight/obese [40]. Living status was reported by the adolescents and grouped in two categories of those living in two-parent families (including married or cohabitating parents or parent/step-parent), and those living in other families such as with their father or mother alone, equally with their mother and father alone, with foster parents or with other guardians. The study sample consisted of 93.5% ethnical Norwegians, and thus differences by ethnicity was not investigated.

### Data analysis

All statistical analyses were performed by IBM® SPSS® Statistics, version 19.0 (IBM Corporation). Descriptive statistics were used to present baseline demographics of adolescents, mothers and fathers. Pearson’s correlation coefficients were conducted to investigate associations of TV/DVD and PC/game time in gender dyads of parents and adolescents at T0 and T2. Differences in adolescents screen time by parental education were obtained by independent-samples *t* test. Attrition analyses were calculated at T0 between the sample of adolescents included in mediation analyses (n=573) and those missing due to incomplete data from mothers’ and/or fathers’ reports (n=402). Independent-samples *t* test was used for continuous variables and Person’s chi-square test was used for categorical variables.

**Figure 1:**
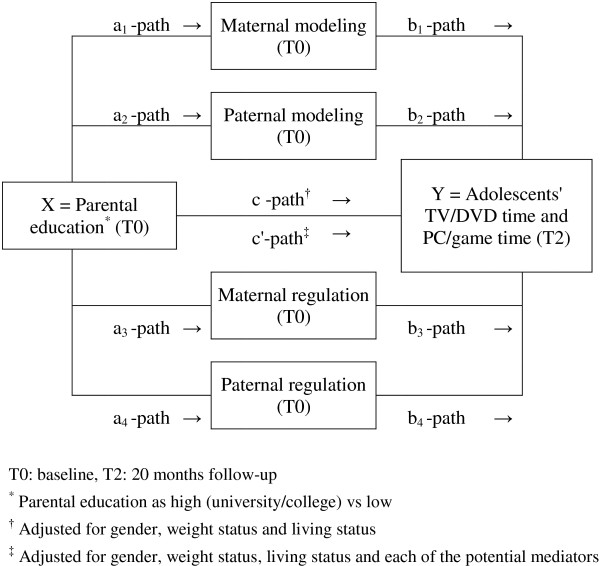
Mediation model of the prediction of adolescents’ screen time.

To investigate the mediation of parental modelling and parental regulation, two models were set up to measure the relationship between parental education and adolescents’ prospective screen time separately for TV/DVD time (model 1) and PC/game time (model 2). Figure 1 illustrates the prediction of parental education at T0 on adolescents’ TV/DVD time (model 1) and PC/game time (model 2) at T2. The possible mediation of parental modelling by TV/DVD time (model 1) and PC/game time (model 2), as measured at T0 by mothers and by fathers, and parental regulation of adolescents’ TV/DVD time (model 1) and PC/game time (model 2), as measured at T0 by mothers and by fathers, was examined.

Cluster effects of adolescents’ screen time have previously been measured in the HEIA cohort, and low unexplained variance (1.5 – 2.8%) was found at the school level [35,36]. The analyses were thus not adjusted for clustering effects by school [41]. Mediation analyses were investigated by linear regression, and unstandardized beta coefficients are presented. In order to control for other possible influences associated with adolescents’ screen time, adolescents’ gender, weight status and living status at T0 were adjusted for in all analyses. To obtain power estimates of 0.8 as recommended in mediation analyses by Fritz and MacKinnon [42], bias-corrected bootstrap analyses were performed using the SPSS script described by Preacher & Hayes [43] with a resampling of 1000 independent samples. Assumptions for the analyses were investigated and considered acceptable.

Single mediation analyses were performed in model 1 and model 2 investigating the total effect of parental education on adolescents’ prospective screen time (c-path) (Figure 1). The relationship between parental education and each of the possible mediating variables were examined at T0 (a-path), as well as the relationship between each of the possible mediating variables and adolescents’ prospective screen time at T2, when adjusted for parental education (b-path) [43,44]. The direct effect of parental education on adolescents’ prospective screen time was then calculated, when adjusted for each of the possible mediating variables of parental modelling and regulation (c’-path). Finally, the mediation effect (a-path x b-path) for each of the possible mediating variables were investigated [43].

## Results

Descriptive statistics of the participants at T0 are shown in Table 1. About 31% of the participants had parents with educational level less than university/college attendance. Adolescents and parents reported similar time spent on TV/DVD, but more time spent on PC/games were generally observed among adolescents than among parents. No significant differences in adolescents’ screen time or any of the correlates were found at T0 between the participants attending both time points (n=908) and those lost to follow up at T2 (n=67) [35]. Attrition analyses between adolescents included in mediation analyses (n=573) and those lost due to missing data in parental reports (n=402) consistently showed no differences in adolescents’ TV/DVD time, but significant less time spent on PC/games were seen among the included adolescents (p=0.01) (data not shown). In the included sample, there was also a significantly larger proportion of adolescents with high level of parental education, normal weight status and living in two-parent families.

**Table 1:** Descriptive statistics of adolescents and parents at baseline (T0)

**Measure**	**Value**	**Boys (n=474)**	**Girls (n=434)**	**Mothers (n=738)**	**Fathers (n=630)**
Age	Mean (SD)	11.2 (0.3)	11.2 (0.3)	40.8 (4.8)	43.4 (5.6)
Weight status^*^	% normal	85.5%	85.4%	-	-
Parental education^†^	% low	30.2%	32.0%	-	-
Living status^‡^	% two-parents	79.2%	81.6%	-	-
TV/DVD (hours/week)	Mean (SD)	12.0 (6.9)	11.1 (6.6)	13.0 (6.3)	13.4 (6.6)
PC/games (hours/week)	Mean (SD)	9.8 (6.7)	7.3 (5.8)	2.6 (3.9)	3.7 (4.5)
TV/DVD regulation^#^	Mean (SD)	-	-	4.1 (1.0)	3.9 (1.0)
PC/game regulation^#^	Mean (SD)	-	-	4.0 (1.0)	3.8 (1.1)

Number of participants (n) varies slightly across variables, SD: Standard Deviation.

^*^ Normal weight vs overweight/obese based on the International Obesity Task Force cut off values.

^†^ Parental education as low vs high (university/college) at T0.

^‡^ Two-parent families vs other families.

^#^ Measured in a five-point Likert scale (from 1=totally disagree to 5=totally agree).

### Screen time behaviours in gender dyads

There were significant associations between mothers and fathers TV/DVD time at T0 with boys and girls TV/DVD time at both time points. Stronger correlations were generally observed at T2 than at T0, except for between fathers and sons reports (Table 2). Moreover, mothers and their sons PC/game time and fathers and their daughters PC/game time were significantly correlated at T0, but no such associations were found at T2.

**Table 2:** Correlation of screen time in gender dyads of parents and adolescents

**Adolescents**		**Mothers (T0)**			**Fathers (T0)**		
			**TV/DVD**	**PC/game**		**TV/DVD**	**PC/game**
**Measure**	**Timepoint**	**n**	**Pearson r**	**Pearson r**	**n**	**Pearson r**	**Pearson r**
TV/DVD (hours/week)							
Boys	Age 11 (T0)	366	0.20***		319	0.15**	
	Age 13 (T2)	369	0.25***		323	0.14*	
Girls	Age 11 (T0)	361	0.18***		304	0.19**	
	Age 13 (T2)	364	0.22***		307	0.30***	
PC/game (hours/week)							
Boys	Age 11 (T0)	362		0.15**	318		0.08
	Age 13 (T2)	367		0.10	321		0.05
Girls	Age 11 (T0)	359		0.09	302		0.12*
	Age 13 (T2)	363		0.08	307		0.10

T0: baseline, T2: 20 months follow-up.

Statistical significance at ^*^p<0.05 ^**^p<0.01 ^***^p<0.001.

### Prediction of adolescents’ screen time by parental education

Significant differences were observed between adolescents’ TV/DVD time and PC/game time at T2 by level of parental education at T0 (Table 3). Single mediation analyses further showed a significant total effect of parental education at T0 on adolescents’ prospective PC/game time at T2 (c-path), where lower parental education was associated with an increase in adolescents PC/game time of 1.4 hours a week (Table 4). A significant direct effect was subsequently observed (p=0.04), after adjusting for either of the possible mediating variables of parental modelling and regulation (c’-path). However, single mediation analyses observed no significant relationship between parental education and adolescents’ prospective TV/DVD time.

**Table 3:** Adolescents’ screen time by parental education

**Screen time (hours/week)**		**Parental education**^**†**^				
			**Low**		**High**	
		**n**	**Mean (SD)**	**n**	**Mean (SD)**	**p-value**^**‡**^
T0	TV/DVD	267	11.9 (7.4)	604	11.3 (6.3)	0.210
	PC/game	266	9.1 (7.0)	599	8.3 (6.0)	0.125
T2	TV/DVD	274	14.2 (7.5)	607	13.0 (7.0)	0.024
	PC/game	274	11.4 (7.8)	605	10.1 (6.8)	0.015

T0: baseline, T2: 20 months follow-up, SD: Standard Deviation.

^†^ Parental education as low vs high (university/college) at T0.

^‡^ Difference between groups of parental education with independent-samples *t* test.

**Table 4:** **Prediction of adolescents’ screen time by single mediation analyses**^**†**^

**Measurement**	**n**	**c-path (SE)**	**a-path (SE)**	**b-path (SE)**	**ab**^**‡**^	**95% CI**
TV/DVD (hours/week)						
Parental education^#^	573	0.75 (0.68)				
Maternal modelling			3.63 (0.55)**	0.28 (0.05)**	1.01	0.62, 1.65
Paternal modelling			3.25 (0.60)**	0.23 (0.05)**	0.75	0.38, 1.27
Maternal regulation			−0.06 (0.10)	−1.40 (0.29)**	0.08	−0.16, 0.42
Paternal regulation			−0.11 (0.10)	−0.60 (0.28)*	0.06	−0.02, 0.33
PC/game (hours/week)						
Parental education^#^	570	1.36 (0.65)*				
Maternal modelling			0.24 (0.36)	0.12 (0.08)	0.03	−0.04, 0.34
Paternal modelling			0.30 (0.41)	0.17 (0.07)*	0.05	−0.07, 0.26
Maternal regulation			0.05 (0.10)	−0.04 (0.27)	0.00	−0.09, 0.05
Paternal regulation			0.05 (0.11)	−0.01 (0.26)	0.00	−0.07, 0.07

SE: Standard Error, CI: Confidence Interval.

Statistical significance at *p>0.05 and **p>0.01.

^†^ Linear regression analysis, adjusted for adolescents’ gender, weight status and living status at baseline.

^‡^ Mediation effects calculated as a-path x b-path.

^#^ Parental education as high (university/college) vs low at baseline.

### Mediation effects of parental modelling and regulation

Significant mediation effects of maternal and paternal modelling at T0 were found on the relationship between parental education at T0 and adolescents’ prospective TV/DVD time at T2 (a-path x b-path) (Table 4). Neither maternal nor paternal regulation mediated the relationship between parental education at T0 and TV/DVD time at T2 in either gender. Mediation effects of parental modelling or regulation were not observed in the relationship between parental education and adolescents’ PC/game time.

### Influence of parental education on parental modelling and regulation

Significant cross-sectional relationships were observed by mediation analyses at T0, between a lower level of parental education and more maternal and paternal TV/DVD time of 3.6 and 3.3 hours a week respectively (a-path, Table#160;4). There was no such relationship between parental education and parental regulation of adolescents’ TV/DVD time. Cross-sectional relationships were neither found between parental education and parental modelling or regulation of adolescents’ PC/game time.

### Prediction of adolescents’ screen time by parental modelling and regulation

Mediation analyses further found more maternal and paternal TV/DVD time at T0 to be significant predictors of more time spent on TV/DVD among adolescents at T2, when adjusted for parental education (b-path). Furthermore, less maternal and paternal regulation at T0 was found to significantly predict more TV/DVD time among adolescents at T2. Paternal modelling of PC/game time was also found to be a significant predictor of adolescents’ time spent on PC/games.

## Discussion

As previously reported in the HEIA cohort study, adolescents’ time spent on TV/DVD significantly increased by 1.4 hours/week among boys and 2.4 hours/week among girls, between the ages of 11 and 13 years [35]. The respective increase in adolescents’ PC/game time was 1.2 and 2.6 hours/week among boys and girls [35]. Significant gender differences were furthermore observed, as boys spent more time on PC/games than girls at both time points [35]. The present study found novel cross-sectional and longitudinal relationships in gender dyads of parents and adolescents’ time spent on TV/DVD and PC/games at the ages of 11 and 13 years. Moreover, both maternal and paternal modelling was found to mediate the prospective relationship between parental education and adolescents’ time spent on TV/DVD.

Correlations observed in gender dyads of parents’ and adolescents’ reports of TV/DVD time are consistent with previous cross-sectional findings of children and adolescents’ TV viewing [28,29,32]. The findings indicate that more time spent on TV/DVD among both parents at the age of 11 years may influence more TV/DVD time among adolescents of both genders at the age of 11 and 13 years. However, a previous study investigating the longitudinal relationship between mothers’, fathers’ and girls’ TV viewing from the age of 9 to 11 years reported no such associations [28]. This may correspond with the present findings of stronger correlation coefficients between parents’ and adolescents’ reports with increasing age. A steady increase of time spent watching TV in gender dyads of parents and adolescents has been reported from the age of 9–10 years to the age of 15–17 years [45]. However, a dip in time was reported between fathers and daughters at the age of 11–12 years [45], which may explain the present findings of a stronger correlation among fathers and daughters’ than among fathers and sons’ in TV/DVD time between the ages of 11 and 13 years in the present study. Previous findings have furthermore reported that parents spend less time with their children in other social contexts as they grow older [45]. The present findings may suggest that adolescents spend more time on TV/DVD with their parents as a shared activity when they grow older, probably as a result of spending time together as a family and due to prolonged waking hours.

The present study furthermore observed cross-sectional associations across genders in dyads of parents’ and adolescents’ PC/game time at the age of 11 years, even though parents generally reported less time spent on PC/games than adolescents during leisure time. Differences in gender dyads of PC/game time may be explained due to large gender differences between girls and boys, and should be further explored. Moreover, parental work time on computers at home was not measured, that may have influenced the results. The evidence on gender relationships between adolescents’ and parents’ use of computer and electronic games is limited. Although, a previous cross-sectional study among Portuguese 7 to 10 year olds showed that paternal TV viewing was significantly related to their daughters’ PC and electronic game time during weekends [32]. In contrast to these findings, reviewed evidence on parent–child relationships generally indicate that mothers invest more time and are more involved in parenting during adolescence compared to fathers, and are thus considered closer to adolescents of both genders [46]. It is important to notice that most previous research has been focusing on the mother as a representative of parents’ and excluded the role of fathers in the home environment [47]. Hence, important relationships may exist between fathers’ and adolescents’ energy balance related behaviours that are currently not understood. Correlations in gender dyads of parents’ and adolescents’ time spent on PC/games were no longer observed at the age of 13 years. The results may reflect the fact that time spent on PC/games is less of a shared activity among parents and adolescents, and thus less influenced by parental behaviours when adolescents grow older. More research is needed in order to identify possible prospective gender specific influences of parental modelling on children’s screen time when growing into adolescence.

Mediation analyses showed a significant total and direct effect between level of parental education and adolescents’ PC/game time from the age of 11 to 13 years, indicating that a lower level of parental education is an important predictor of more time spent on PC/games among adolescents. No prospective relationship was observed in mediation analysis of parental education on adolescents’ TV/DVD time between the ages of 11 to 13 years. A previous study reported inconsistent results in the prospective relationship between parental education at the age of 13 years and girls’ TV viewing and videogame playing after 6 months [48], and no relationships were reported between parental education level at the age of 9–12 years and adolescents’ TV viewing, playing video games or computer use at the age of 14 years and older [49]. The lack of influence from parental education on adolescents’ prospective TV/DVD time in the present study may be caused by the fact that social differences in adolescents’ prospective TV/DVD time are less important among older adolescents. Hence, other determinants in the home may be stronger in influencing adolescents’ prospective behaviour, such as parental modelling, regulation, availability and accessibility of TV in the home as well as presence and interaction with siblings. However, the level of parental education was high in this group of 11 year olds, and analyses were based on a binominal variable which may not capture all gradients of parental education. More research is needed in order to investigate the prospective relationship between parental education and different screen time behaviours during adolescence.

Maternal and paternal modelling significantly mediated the prospective relationship between parental education and adolescents’ TV/DVD time. However, no mediation was found by parental regulation of adolescents’ prospective TV/DVD time, as differences in regulation by parental education were not found. A recent cross-sectional study found number and placement of TV in the home to be the strongest mediators to the relationship between maternal education and 11 year old children’s TV viewing [25]. Parental behaviour was not investigated as a potential mediator, but restrictions on how much time the child spent on TV viewing was shown to be less important as a mediator among 11 year olds than among 6 year olds [25]. The present study did neither find parental modelling nor regulation to mediate the relationship of parental education on adolescents’ prospective PC/game time. These results imply that parental modelling is more important as an intermediate variable when explaining differences by parental education in adolescents’ prospective TV/DVD time than in time spent on PC/games. The results may indicate that adolescents’ are more independent in their leisure time using PC/games, than what is observed for watching TV/DVD. Moreover, time spent on PC/games may be more reflected by social contexts with siblings and friends rather than with parents. Other factors in the home environment may thus be important mediators of adolescents’ time spent on PC/games by parental education. In addition to parental modelling and regulation, determinants such as availability and accessibility of computers in the home, presence and interaction with siblings and friends should be a matter for further investigation. These could be important modifiable determinants to target in interventions aiming to reduce social inequalities in adolescents’ PC/game time by parental education.

Both maternal and paternal regulation at the age of 11 years was significantly related to less time spent on TV/DVD among 13 year old adolescents (b-path). The results imply the importance of parental regulation in order to reduce adolescents’ prospective TV/DVD time, independently of parental education. This may thus be an important determinant to target in order to reduce adolescents’ prospective TV/DVD time. No such relationships were seen for parental regulation of adolescents PC/game time. A recent review concluded consistently with an inverse cross-sectional association between parental rules and adolescents’ screen time, where most of the studies investigated adolescents’ TV viewing [26]. An inverse longitudinal relationship was furthermore reported between perceived presence of family rules and TV viewing among adolescents over 2 years [50]. It is important to notice that parental regulation was only measured with a single question item in the present study, and may therefore not capture all facets of parental rules of TV/DVD and PC/game time.

The results should be viewed in light of some limitations of the study. Although the participation rate of sampled schools was low (21%), there was no significant difference between schools who participated in the study and schools that declined participation in terms of number of students in the 6^th^ grade and overall size [51]. However, socioeconomic characteristics of schools were not measured, as such data is not nationally available in Norway. The outcome measures were self-reported, which are associated with problems of misreporting of adolescents screen time, due to issues of social expectations and norms [52]. Although, adolescents’ ability to self-report are considered to be fully developed at this age [53]. The test-retest correlation results of the outcome measures were borderline in the present study, thus the reliability of the results may be questioned. However, the larger sample size the larger tolerance of a less reliable instrument, and thus we believe the reliability of these measurements to be acceptable [54]. Attrition analysis showed no significant differences in adolescents’ baseline TV/DVD time between those included and those excluded from the analyses. However, significant differences were observed in adolescents’ PC/game time, weight status, living status and level of parental education. This may indicate that the included participants were more health conscious than the rest of the population.

Strengths of the present study were the longitudinal study design based on a relatively large sample size at a narrow age-range, with high participation rate over time. Information on parental education, modelling and regulation was collected through parental reports, which is considered to give more reliable measurements than when reported by the adolescents [55]. Moreover, measurements of multiple screen time behaviours and their determinants were included.

## Conclusion

The present study found that both parents’ TV/DVD time is associated with adolescents’ TV/DVD time in both genders at the age of 11 years, and between the ages of 11 to 13 years. Opposite gender dyads were observed for PC/game time at the age of 11 years, but the association was not seen in either gender between the ages of 11 and 13 years. Further investigations of different screen time behaviours in gender dyads of both parents and their children should be emphasized. Parental TV/DVD time was further found to mediate the relationship between parental education and adolescents’ prospective TV/DVD time across genders. Hence, parental modelling are important to consider in interventions aiming to reduce social differences by parental education in the prevention of adolescents’ prospective time spent on TV/DVD. Other factors in the home environment may influence adolescents’ prospective screen time and should be included in future studies, such as the availability and accessibility of screens in the home.

## Abbreviations

HEIA: The Norwegian HEalth In Adolescents study; TV/DVD: television and video viewing; PC/game: computer and electronic game use.

## Competing interests

This work was supported by the Norwegian Extra Foundation for Health and Rehabilitation through the National Association of Public Health. The HEIA study was originally funded by the Norwegian Research Council [grant number 155323/V50] with supplementary funds from the Throne Holst Nutrition Research Foundation, the University of Oslo and the Norwegian School of Sport Sciences. The authors THT, MB, NL, IHB, MKG, MG, YO and LFA declare that they have no competing interests.

## Authors’ contributions

THT drafted the first manuscript, conducted the statistical analyses and revised the paper based on the comments by the other co-authors. MB, NL, IHB, MG, YO and LFA participated in designing the study, project planning and/or data collection. All authors have critically read and revised the paper, and approved the final manuscript.
